# Editorial: Therapeutic effects of endogenous hormones in pathologies linked to metabolic and/or inflammatory disorders

**DOI:** 10.3389/fphar.2025.1699758

**Published:** 2025-09-23

**Authors:** Sandra Feijóo-Bandín, Alana Aragón-Herrera

**Affiliations:** ^1^ Cellular and Molecular Cardiology Research Unit, Instituto de Investigación Sanitaria de Santiago de Compostela (IDIS) , Complexo Hospitalario Universitario de Santiago de Compostela, Área Sanitaria Santiago de Compostela e Barbanza (SERGAS), Santiago de Compostela, Spain; ^2^ Centro de Investigación Biomédica en Red de Enfermedades Cardiovasculares (CIBERCV), Instituto de Salud Carlos III, Madrid, Spain

**Keywords:** hormones, inflammation, metabolism, human diseases, therapy

Advances in the study of biological pathways regulated by hormones and their mechanisms of action have paved the way for promising research into the development of new hormone-based medicines. These drugs are highly desirable as a therapeutic option due to their unique characteristics, including high target specificity, well-defined physiological activities in the body, efficacy, and safety (Wang et al., 2022). Some endogenous hormones have been extensively used in clinical practice and are well-established, such as insulin, calcitonin, and the adrenocorticotropic hormone. Initially, these hormones were isolated from natural sources; however, subsequent biotechnology breakthroughs enabled their production as synthetic or recombinant agents, resulting in reduced costs, increased market availability, and the possibility of introducing modifications in their structure to overcome stability and bioavailability issues (Lau and Dunn, 2018). Currently, around 100 hormone-based therapeutic agents have been approved for use in different countries (Xiao et al., 2025), highlighting their effectiveness and the importance of continued investigation to expand the possibilities of developing new hormonal therapies for treating human diseases.

Metabolism and inflammation are two fundamental biological processes that are essential for survival. Metabolism controls energy utilisation, while inflammation handles defence and repair, both responding to stressors to restore the organism’s homeostasis in such a way that their balance and interplay are crucial in managing health and disease (Hotamisligil, 2006). Nowadays, it is widely accepted that understanding and targeting metabolism and inflammation are vital for developing effective therapeutic approaches for pathologies in which the deregulation of these processes contributes to the development of the disease (Hu et al., 2024). In this line, many hormones and/or their analogs have already been approved for use or are being tested in clinical trials (Xiao et al., 2025); however, more research is still needed.

Due to the relevance of understanding the potential therapeutic effects of endogenous hormones in pathologies linked to metabolic and/or inflammatory disorders, the current issue of Frontiers in Pharmacology includes a series of six articles on this Research Topic:

Zhu et al. describe a new effect of dehydroepiandrosterone, a steroid hormone produced by the adrenal glands that is a crucial precursor for sex hormones, as a cardioprotective agent by the activation of the estrogen receptors through the triggering of C-Jun N-terminal Kinase pathway to prevent endoplasmic reticulum stress mediated apoptosis in human vascular smooth muscle cells and human umbilical vein endothelial cells, which could help maintain a normal vascular function and prevent the development/worsening of cardiovascular diseases, usually linked to metabolic and inflammatory derangements (Taube et al., 2012).

El-Sherbiny et al. describe how oxytocin, a neuro-pituitary hormone with main effects on labor, milk expulsion during breastfeeding, and social bonding behaviors, can alleviate testicular dysfunction induced by lipopolysaccharide in rats by reducing oxidative damage and inflammation in the testicular tissue as well as reestablishing the disrupted sperm count, motility, and morphology. These outcomes propose that oxytocin could be a novel therapeutic strategy for male infertility and testicular dysfunction occurring from acute inflammatory disorders.

Ruiz-Pozo et al. present a review article on the role of cortisol, melatonin, thyroid hormones, sex hormones, and insulin on the modulation of the immune response to human metapneumovirus (hMPV) infection, and comment on the possible therapeutic approaches that could be developed for the treatment of hMPV based on the signaling pathways of these hormones.

Habek et al. show for the first time that uroguanylin, a hormone secreted by the duodenum that regulates the interplay of gut and brain for the homeostasis of food intake and energy metabolism, is not only delivered by the circulation into the brain but also produced in the brain of both mice and humans, being its expression regulated by feeding. Moreover, they describe that acute and chronic brain-derived uroguanylin increase brown adipose tissue activity and volume and improve glucose homeostasis in mice and suggest the chronic central administration of uroguanylin as a new therapeutic approach for improving postprandial glucose regulation in patients with type 2 diabetes mellitus.

Della Corte et al. present a pilot study regarding the potential use of urodilatin, a natriuretic hormone produced in the renal distal tubules, as a new diagnostic marker for renal salt wasting syndrome, whose symptoms and laboratory characteristics overlap with the syndrome of inappropriate antidiuretic hormone secretion, making difficult its clinical management.

Finally, Ramos-Zavala et al. describe that netrin-1, a secreted peptide initially identified as a chemotropic guidance to vertebrate development, and adiponectin, a classical hormone implicated in the regulation of energy metabolism, exhibit opposing serum concentrations in individuals with different metabolic profiles. Besides, the authors describe a new role of netrin-1 as a pro-inflammatory molecule involved in the retention of macrophages in the adipose tissue, contributing to the pathophysiology of obesity-related metabolic dysfunction and insulin resistance, with adiponectin exerting the opposite effect, and suggest the combination of netrin-1 and adiponectin plasma levels determination as a better therapeutic approach to diagnose and monitor metabolic diseases.

Together, these six works illustrate the therapeutic possibilities of unraveling and understanding the mechanisms of action of hormones, new and previously described, in different scenarios related to metabolic and inflammatory disorders to take advantage of their properties and use them as safe and effective drugs. With this Research Topic, we aimed to put in the spotlight the valuable benefits for the population of exploiting this field of research, and to motivate our readers to contribute to the development of this area.
